# Docosahexaenoic Acid Inhibits UVB-Induced Activation of NF-κB and Expression of COX-2 and NOX-4 in HR-1 Hairless Mouse Skin by Blocking MSK1 Signaling

**DOI:** 10.1371/journal.pone.0028065

**Published:** 2011-11-28

**Authors:** Md. Mostafizur Rahman, Joydeb Kumar Kundu, Jun-Wan Shin, Hye-Kyung Na, Young-Joon Surh

**Affiliations:** 1 Tumor Microenvironment Research Center, College of Pharmacy, Seoul National University, Seoul, South Korea; 2 Department of Food and Nutrition, Sungsin Women's University, Seoul, South Korea; 3 WCU Department of Molecular Medicine and Biopharmaceutical Sciences, Graduate School of Convergence Science and Technology, Seoul National University, Seoul, South Korea; 4 Cancer Research Institute, Seoul National University, Seoul, South Korea; The University of Texas M. D. Anderson Cancer Center, United States of America

## Abstract

Exposure to ultraviolet-B (UVB) radiation induces inflammation and photocarcinogenesis in mammalian skin. Docosahexaenoic acid (DHA), a representative ω-3 polyunsaturated fatty acid, has been reported to possess anti-inflammatory and chemopreventive properties. In the present study, we investigated the molecular mechanisms underlying the inhibitory effects of DHA on UVB-induced inflammation in mouse skin. Our study revealed that topical application of DHA prior to UVB irradiation attenuated the expression of cyclooxygenase-2 (COX-2) and NAD(P)H:oxidase-4 (NOX-4) in hairless mouse skin. DHA pretreatment also attenuated UVB-induced DNA binding of nuclear factor-kappaB (NF-κB) through the inhibition of phosphorylation of IκB kinase-α/β, phosphorylation and degradation of IκBα and nuclear translocation of p50 and p65. In addition, UVB-induced phosphorylation of p65 at the serine 276 residue was significantly inhibited by topical application of DHA. Irradiation with UVB induced phosphorylation of mitogen and stress-activated kinase-1 (MSK1), extracellular signal-regulated kinase (ERK) and p38 mitogen-activated protein (MAP) kinase, and all these events were attenuated by pretreatment with DHA. Blocking ERK and p38 MAP kinase signaling by U0126 and SB203580, respectively, diminished MSK1 phosphorylation in UVB-irradiated mouse skin. Pretreatment with H-89, a pharmacological inhibitor of MSK1, abrogated UVB-induced activation of NF-κB and the expression of COX-2 and NOX-4 in mouse skin. In conclusion, topically applied DHA inhibits the UVB-induced activation of NF-κB and the expression of COX-2 and NOX-4 by blocking the phosphorylation of MSK1, a kinase downstream of ERK and p38 MAP kinase, in hairless mouse skin.

## Introduction

Ultraviolet B (UVB) radiation is the most prevalent environmental carcinogen that increases the risk of skin cancer [1]. Oxidative stress and persistent inflammation are the key pathologic events in UVB-induced skin photocarcinogenesis [2]. NAD(P)H:oxidases (NOX), a family of inducible membrane bound and cytosolic enzymes, is involved in the generation of reactive oxygen species (ROS) [3]. The expression and activity of different isoforms of NOX are elevated in various human cancers [4], [5]. NOX-4, a member of the NOX family proteins, is an oncoprotein [6] that contributes to the transformation, proliferation and migration of cancer cells [7], [8]. Although NOX is involved in UVB-induced generation of ROS in human keratinocytes [9], it is yet to be investigated if UVB irradiation can induce NOX-4 expression in mouse skin in vivo.

Cyclooxygenase-2 (COX-2), a rate limiting enzyme in the biosynthesis of prostaglandins, has been implicated in carcinogenesis [10]. Elevated expression of COX-2 has been documented in hyperplastic skin, benign papillomas and squamous cell carcinomas of UVB-irradiated mouse skin [11]. The increased susceptibility of *cox-2* transgenic mice to chemically induced skin papillomagenesis [12] and the reduced incidence and the multiplicity of skin tumors in *cox-*2 knockout mice support the role of COX-2 in skin carcinogenesis [13]. Moreover, pharmacological inhibition of COX-2 protected against UVB-induced mouse skin tumorigenesis [14]. Notably, UVB irradiation induces the expression of COX-2 in mouse skin through inappropriate amplification of cell signaling pathways consisting of various kinases and their downstream transcription factors [15], [16], [17].

Nuclear factor-kappaB (NF-κB), a heterodimer of p65 and p50 proteins, is a redox-sensitive transcription factor which plays a key role in COX-2 expression in mouse skin upon irradiation with UVB [18]. Exposure to UVB radiation leads to the phosphorylation of mitogen-activated protein (MAP) kinases, such as extracellular signal-regulated kinase (ERK) [19] and p38 MAP kinase [15], [17], which activates NF-κB and induces COX-2 expression [20]. The activation of p38 MAP kinase and NF-κB is also involved in the induction of NOX-4 expression in γ-radiation-stimulated lung fibroblasts [21] and tumor necrosis factor-α (TNFα)-treated human aortic smooth muscle cells [22], respectively. A common substrate for ERK and p38 MAP kinase is mitogen- and stress-activated kinase-1 (MSK1), which transmits signals down to NF-κB in hydrogen peroxide-stimulated skeletal myoblasts [23]. However, the role of MSK1 in UVB-irradiated mouse skin inflammation is yet to be investigated. This prompted us to examine the role of MSK1 in the UVB-induced activation of NF-κB and expression of COX-2 and NOX-4 in mouse skin.

Since COX-2 and NOX-4 play important roles in inducing oxidative stress and inciting inflammation, targeted inhibition of signaling pathways associated with the aberrant expression of these proteins would be a rational approach for chemoprevention [24]. Docosahexaenoic acid (DHA), a representative ω-3 polyunsaturated fatty acid abundantly present in fish oil and some plant seed oils, possesses antioxidative, anti-inflammatory and chemopreventive properties [25]. However, the effects of DHA on UVB-induced mouse skin inflammation and its underlying molecular mechanisms have not been investigated yet. Here, we report that topical application of DHA inhibits UVB-induced expression of COX-2 and NOX-4 in mouse skin by blocking the activation of NF-κB through the inhibition of ERK- and p38 MAP kinase-mediated phosphorylation of MSK1.

## Results

### DHA inhibits UVB-induced expression of COX-2 and NOX-4 in HR-1 hairless mouse skin

**Figure 1:**
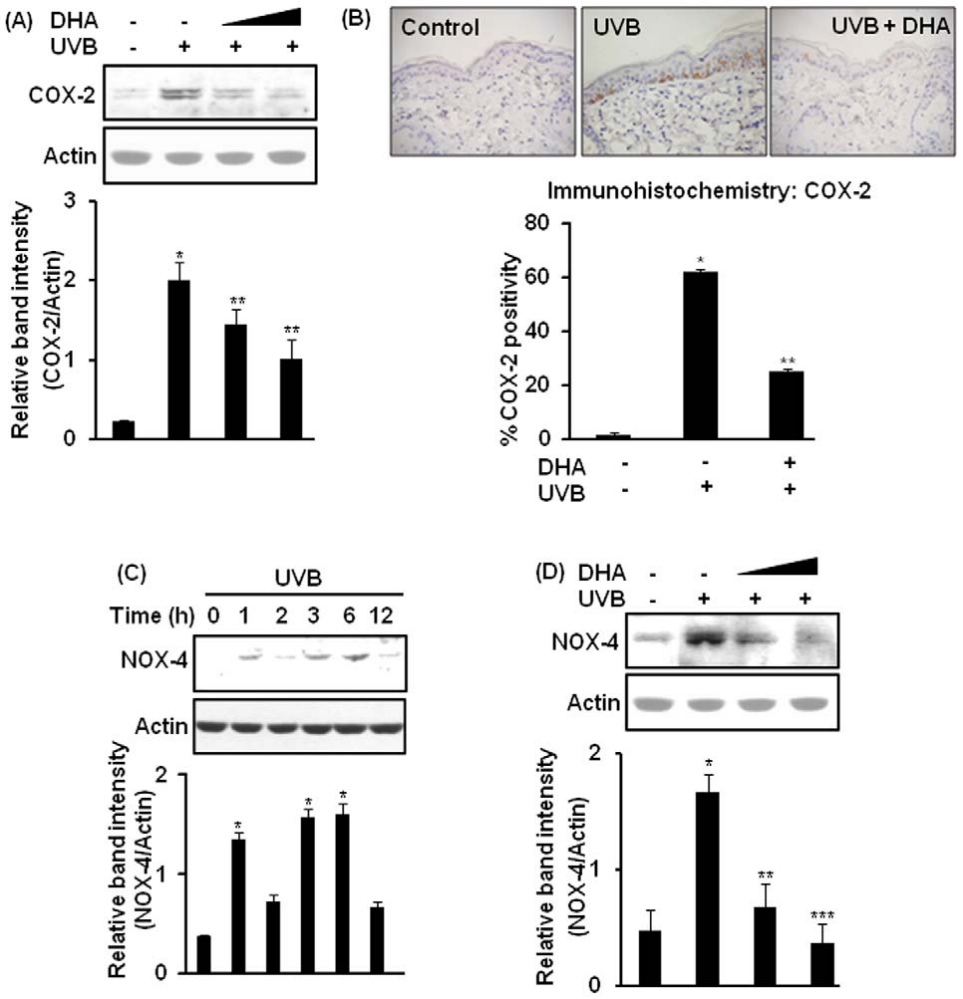
Inhibitory effects of DHA on UVB-induced expression of COX-2 and NOX-4 in mouse skin. Dorsal skin of female HR-1 hairless mice (n = 3 per treatment group) were treated topically with DHA (2.5 or 10 µmol/mouse) dissolved in 0.2 ml acetone 40 min before UVB (180 mJ/cm^2^) irradiation, and sacrificed after 6 h. Control animals were treated with acetone and left unirradiated. (**A**) Epidermal lysates were analyzed for COX-2 expression by immunoblotting. *, *p*<0.001 (control versus UVB); **, *p*<0.01 (UVB only versus 2.5 or 10 µmol DHA plus UVB). (**B**) Skin samples from mice treated as above were subjected to immunohistochemical analysis by using affinity purified murine COX-2 antibody as described in Materials and Methods. Positive COX-2 staining yielded a brown-colored product: acetone (left), UVB alone (middle), and UVB plus 10 µmol DHA (right). Percent of COX-2 positivity was calculated as the ratio of number of COX-2-stained cells to total number of epidermal cells counted from 10 equal sections of immunostained tissues from each animal. *, *p*<0.001 (control versus UVB); **, *p*<0.01(UVB versus 10 µmol DHA plus UVB). (**C**) Dorsal skin was irradiated with UVB and animals were sacrificed at indicated time periods for assessing NOX-4 expression. *, *p*<0.001 (control versus UVB). (**D**) Mice were treated with DHA and irradiated with UVB as indicated above and epidermal NOX-4 expression was examined by immunoblotting. *, *p*<0.001 (control versus UVB); **, *p*<0.01 (UVB alone versus 2.5 µmol DHA plus UVB); ***, *p*<0.001 (UVB alone versus 10 µmol DHA plus UVB).

The expression of COX-2 is transiently induced by diverse stimuli including exposure to UVB radiation [15], [18]. We have previously reported that irradiation with UVB (180 mJ/cm^2^) induces COX-2 expression in HR-1 hairless mouse skin maximally at 6 h [26]. In the present study, pretreatment with DHA (2.5 or 10 µmol) significantly attenuated UVB-induced COX-2 expression in mouse skin at 6 h post-irradiation (Fig. 1A). Immunohistochemical analysis further confirmed the inhibitory effect of DHA on UVB-induced epidermal COX-2 expression (Fig. 1B). Further analysis of immunohistochemical data revealed that DHA significantly decreased the proportion of epidermal cells expressing COX-2. NOX-4, a superoxide generating enzyme, is an oncoprotein [6] that is overexpressed in melanoma cells [8]. We examined the NOX-4 expression level in UVB-irradiated mouse skin. As shown in Fig. 1C, exposure to UVB radiation induced NOX-4 expression in HR-1 hairless mouse skin in a time-dependent fashion with maximum induction at 6 h post-irradiation. Topical application of DHA onto mouse skin prior to UVB irradiation significantly reduced NOX-4 expression (Fig. 1D).

### DHA attenuates UVB-induced activation of NF-κB in hairless mouse skin

**Figure 2:**
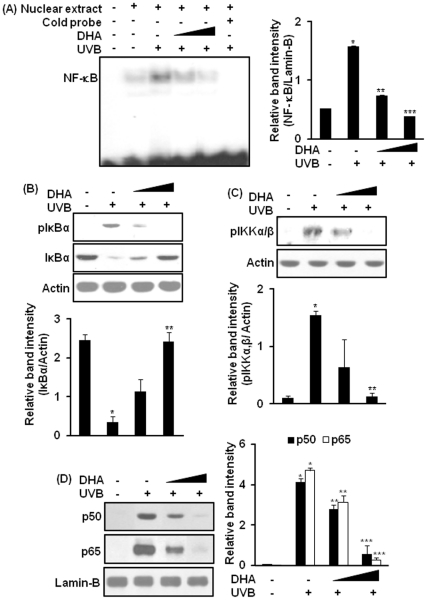
DHA inhibits UVB-induced activation of NF-κB in mouse skin. Female HR-1 hairless mice (n = 3 per treatment group) were treated topically with DHA (2.5 or 10 µmol/mouse) dissolved in 0.2 ml acetone. After 40 min, animals were exposed to UVB radiation (180 mJ/cm^2^). Control animals were treated with acetone and were not exposed to UVB. Animals were sacrificed 1.5 h post-UVB irradiation. The epidermal nuclear extract was prepared as described in Materials and Methods and analyzed by the EMSA. (**A**) Effects of DHA on UVB-induced NF-κB DNA binding. Representative data showing probe only (lane 1), vehicle control (lane 2), UVB alone (lane 3), 2.5 µmol DHA plus UVB (lane 4), 10 µmol DHA plus UVB (lane 5), and nuclear extract from UVB-treated skin plus 100-fold excess unlabeled NF-κB oligonucleotide (lane 6). *, *p*<0.001 (vehicle only versus UVB); **, *p*<0.01 (UVB versus 2.5 µmol DHA plus UVB); ***, *p*<0.001 (UVB versus 10 µmol DHA plus UVB). (**B**) Cytosolic extract was prepared as described in Materials and Methods and subjected to Western blot analysis to determine the expression of total and phosphorylated IκBα. *, *p*<0.001 (control versus UVB); **, *p*<0.001 (UVB versus 10 µmol DHA plus UVB). (**C**) Epidermal lysate was analyzed for phosphorylation of IKKα/β (serine 181) by immunoblot analysis. *, *p*<0.001 (control versus UVB); **, *p*<0.001 (UVB versus 10 µmol DHA plus UVB). (**D**) Epidermal nuclear extract was assayed for nuclear localization of p50 and p65. *, *p*<0.001 (control versus UVB); **, *p*<0.05 (UVB versus 2.5 µmol DHA plus UVB); ***, *p*<0.001 (UVB versus 10 µmol DHA plus UVB).

The promoter region of murine *cox-2* gene harbors binding sites for NF-κB [27]. Moreover, the transcriptional activation of NOX-4 is regulated in part by NF-κB in TNFα-stimulated human aortic smooth muscle cells [22]. We have previously reported that UVB radiation activates NF-κB in hairless mouse skin at 1 h which persists until 6 h post-irradiation [19]. Since NF-κB is involved in the transcriptional activation of COX-2 [27] and NOX-4 [22], we examined the effect of DHA on NF-κB activation in UVB-irradiated hairless mouse skin. As illustrated in Fig. 2A, pretreatment with DHA (2.5 or 10 µmol) inhibited UVB-induced DNA binding of NF-κB in mouse skin. Topical application of DHA significantly diminished the phosphorylation and degradation of IκBα (Fig. 2B), phosphorylation of IKKα/β (Fig. 2C), and subsequent nuclear translocation of p65 and p50 proteins (Fig. 2D) in UVB-irradiated mouse skin.

### DHA inhibits phosphorylation of MSK1 by blocking the activation of ERK and p38 MAP kinase in UVB-irradiated mouse skin

**Figure 3:**
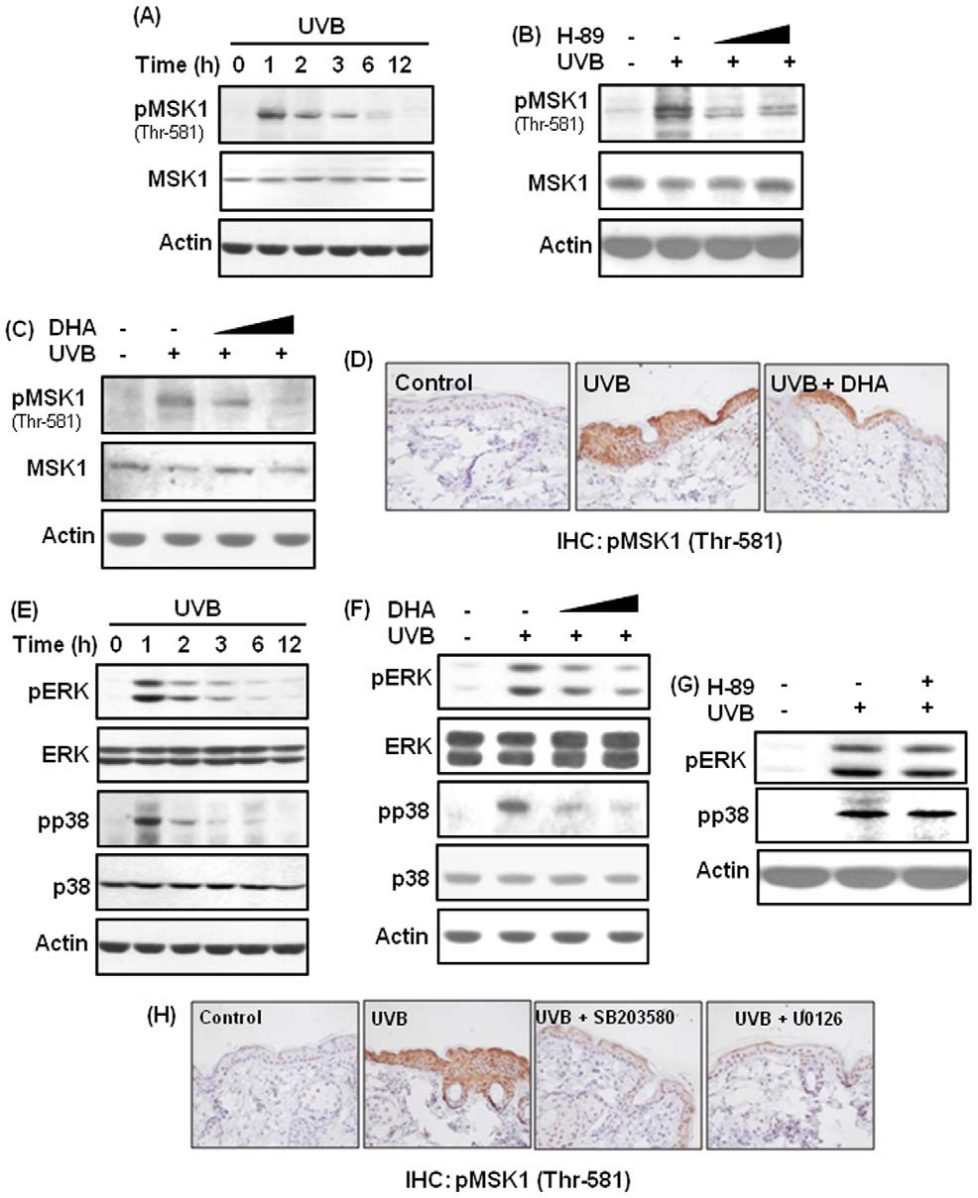
DHA inhibits UVB-induced phosphorylation of MSK1 by blocking ERK and p38 MAP kinase in mouse skin. (**A,E**) Mice (n = 3 per group) were irradiated with UVB (180 mJ/cm^2^) and sacrificed at indicated time periods. (**A**) Tissue lysates were separated by 10% SDS-polyacrylamide gel, transferred to PVDF membrane and immunoblotted to detect the expression of phosphorylated (threonine 581) MSK1. **Figure S1A** represents the statistical analysis of image density of UVB-induced expression of phospho-MSK1. (**B–D, F–H**) Animals were treated topically with H-89 (25 or 100 nmol/mouse), DHA (2.5 or 10 µmol/mouse), U0126 (5 µmol/mouse) or SB203580 (5 µmol/mouse) 40 min before exposure to UVB and were sacrificed 1.5 h post-irradiation. (**B**) Epidermal lysates from mice irradiated with UVB in presence or absence of H-89 (25 or 100 nmol/mouse) were immunoblotted for detecting the expression of MSK1 and phospho-MSK1. **Figure S1B** represented the densitometric analysis of immunoblots. (**C**) Epidermal protein extracts from mice irradiated with UVB with or without pretreatment with DHA (2.5 or 10 µmol) were subjected to Western blot analysis to detect the expression of MSK1 and pMSK1 (threonine 581). **Figure S1C** illustrated the quantification of band intensity of phospho-MSK1. (**D**) Immunohistochemical analysis of pMSK1 expression in mouse epidermis upon irradiation with UVB in presence or absence of DHA. (**E**) Epidermal lysates were subjected to Western blot analysis to examine the expression of phosphorylated ERK and p38 MAP kinase upon UVB irradiation. Quantification and statistical analysis of immunoblots were presented in **Figure S1D**. (**F**) Inhibitory effects of DHA on UVB-induced phosphorylation of ERK and p38 MAP kinase. **Figure S1E** represents the quantification data of phospho-ERK and phospho-p38 MAP kinase. (**G**) Effect of H-89 on the phosphorylation of ERK and p38 MAP kinase in UVB-irradiated mouse skin. Assessment of relative band intensity was presented in **Figure S1F**. (**H**) Skin samples from mice pretreated with SB203580 or U0126 (each 5 µmol in 0.2 ml acetone) and irradiated with UVB were subjected to immunohistochemical analysis. Positive phospho-MSK1 staining yielded a brown-colored product.

A panel of upstream serine/theronine kinases transmit activating signal to NF-κB. MSK1 is a serine/threonine kinase known to induce NF-κB activation in response to diverse stimuli, such as interleukin-1β (IL-1β) [28] and TNFα [29]. Moreover, MSK1 is a substrate for ERK and p38 MAP kinase [23], which regulate UVB-induced activation of NF-κB in keratinocytes [30] and in SKH hairless mouse skin [31]. However, it is yet to be examined if MSK1 is phosphorylated in mouse skin upon UVB exposure. We found that exposure of HR-1 hairless mouse skin to UVB radiation led to the phosphorylation of MSK1 (threonine 581) at 1 h (Fig. 3A, Fig. S1A). The UVB-induced phosphorylation of MSK1 was inhibited by pretreatment with H-89, a pharmacological inhibitor of MSK1 (Fig. 3B, Fig. S1B). Pretreatment with DHA also diminished UVB-induced phosphorylation of MSK1 in mouse skin as revealed by immunoblot (Fig. 3C, Fig. S1C) as well as immunohistochemical analyses (Fig. 3D). We also found that exposure to UVB radiation induced the phosphorylation of ERK and p38 MAP kinase in mouse skin at 1 h post irradiation (Fig 3E, Fig. S1D), and this was attenuated by pretreatment with DHA (Fig. 3F, Fig. S1E). Topical application of H-89 at a dose of 25 nmol that inhibited MSK1 phosphorylation failed to diminish UVB-induced phosphorylation of ERK and p38 MAP kinase (Fig. 3G, Fig. S1F). On the other hand, pretreatment with U0126 (5 µmol) and SB203580 (5 µmol), pharmacological inhibitors of ERK and p38 MAP kinase, respectively, attenuated UVB-induced MSK1 phosphorylation in mouse skin (Fig. 3H).

### A role of MSK1 in UVB-induced activation of NF-κB in mouse skin and its modulation by DHA

**Figure 4:**
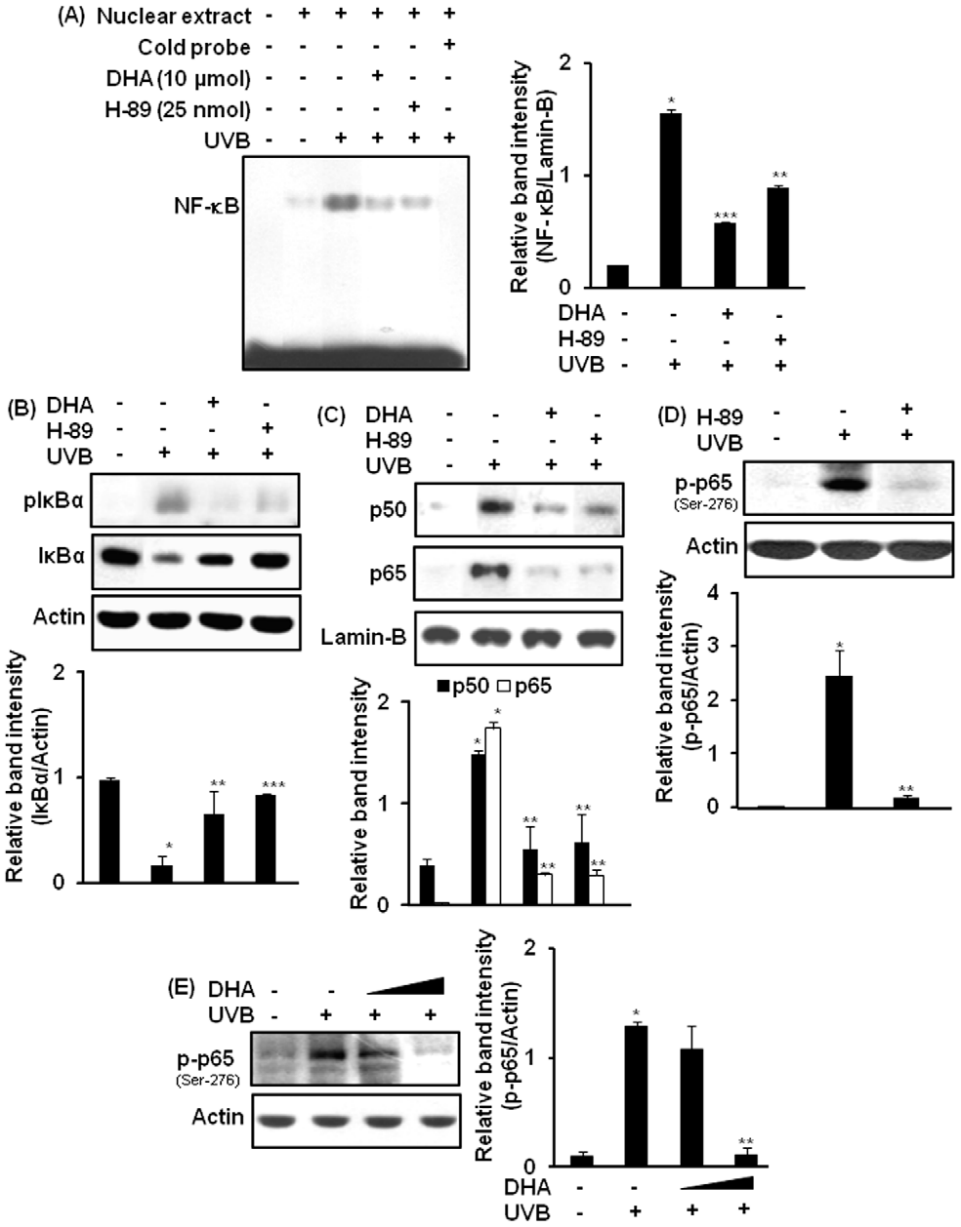
Inhibitory effect of DHA on UVB-induced activation of NF-κB is mediated through inhibition of MSK1. Mice (n = 3 per treatment group) were treated topically with H-89 (25 nmol) or DHA (10 µmol) as described in figure 2 and sacrificed 1.5 h later. (**A**) Nuclear protein (10 µg) was subjected to EMSA to examine the effect of H-89 or DHA on UVB-induced DNA binding of NF-κB. *, *p*<0.001 (control versus UVB); **, *p*<0.01 (UVB versus 10 µmol DHA plus UVB); ***, *p*<0.001 (UVB versus 25 nmol H-89 plus UVB). (**B**) Inhibitory effects of H-89 or DHA on UVB-induced phosphorylation of IκBα was measured by immunoblot analysis. *, *p*<0.001 (control versus UVB); **, *p*<0.01 (UVB versus 10 µmol DHA plus UVB); ***, *p*<0.001 (UVB versus 25 nmol H-89 plus UVB). (**C**) Nuclear extracts were analyzed by western blotting for nuclear translocation of p50 and p65. *, *p*<0.001 (control versus UVB); **, *p*<0.01 (UVB versus 10 µmol DHA or 25 nmol H-89 plus UVB). (**D**) Dorsal skin of HR-1 hairless mice was treated topically with H-89 (25 nmol/mouse) followed by irradiation with UVB to detect the phosphorylation of NF-κB p65 (serine-276). *, *p*<0.001 (control versus UVB); **, *p*<0.001 (UVB versus 25 nmol H-89 plus UVB). (**E**) Epidermal lysates from mice pretreated with DHA (2.5 or 10 µmol/mouse) and irradiated with UVB were subjected to western blotting analysis to determine the phosphorylation of NF-κB p65 (serine-276). *, *p*<0.001 (control versus UVB); **, *p*<0.001 (UVB versus 10 µmol DHA plus UVB). Figure S2, represents the effect of H-89 on UVB-induced phosphorylation of IKKα/β (Ser 181).

We examined whether pharmacological inhibition of MSK1 could suppress the activation of NF-κB in HR-1 hairless mouse skin. Irradiation of dorsal skin of hairless mice with UVB induced the DNA binding of NF-κB, which was abrogated by pretreatment with H-89 (Fig. 4A). H-89 (25 nmol) also inhibited UVB-induced phosphorylation and subsequent degradation of IκBα (Fig. 4B), but failed to inhibit phosphorylation of IKKα/β (Fig. S2). In addition, treatment of mouse skin with H-89 attenuated nuclear translocation of p50 and p65/RelA in UVB-irradiated mouse skin **(** Fig. 4C **)**. The phosphorylation of p65 at the serine 276 residue in UVB-irradiated mouse skin was also inhibited by pretreatment with either H-89 (Fig. 4D) or DHA (Fig. 4E).

### Involvement of MSK1 in UVB-induced expression of COX-2 and NOX-4 in mouse skin

**Figure 5:**
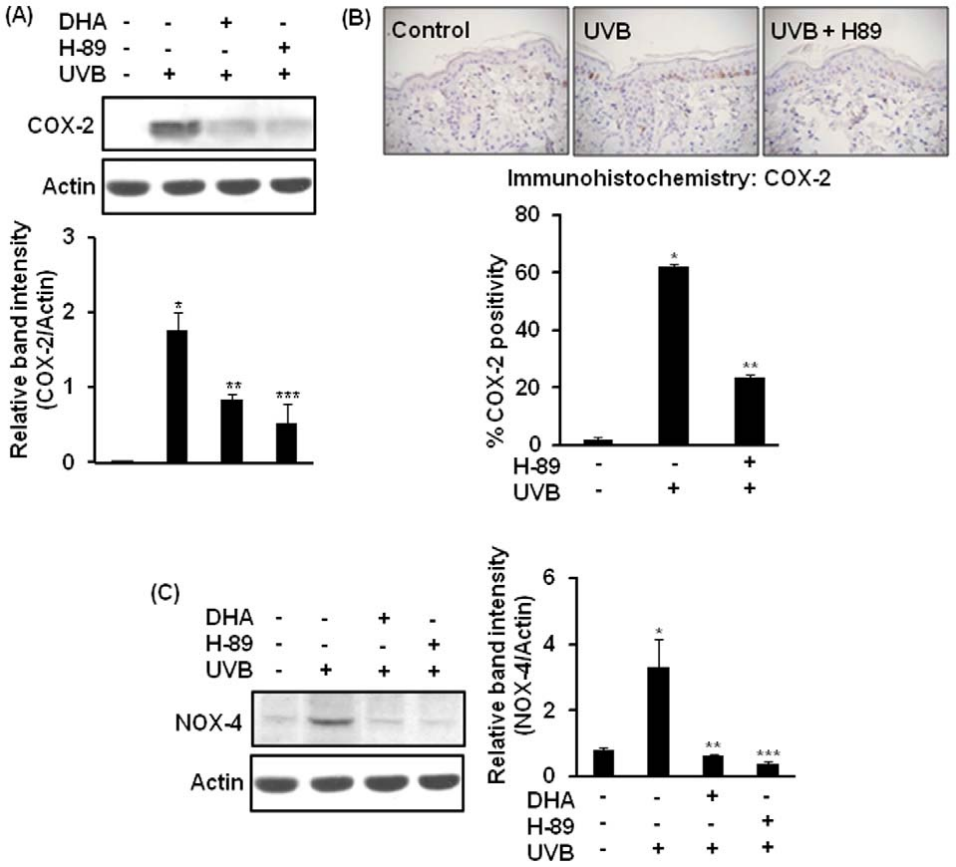
Role of MSK1 in UVB-induced expression of COX-2 and NOX-4 in mouse skin. Animals were treated topically with H-89 (25 nmol) or DHA (10 µmol) as described in Fig. 1 and sacrificed 6 h post irradiation. (**A**) Effect of H-89 or DHA on UVB-induced COX-2 expression was analyzed by Western blot analysis. *, *p*<0.001 (control versus UVB); **, *p*<0.01 (UVB versus 10 µmol DHA plus UVB); ***, *p*<0.01 (UVB versus 25 nmol H-89 plus UVB). (**B**) Formalin-fixed skin tissues pretreated with H-89 and exposed to UVB radiation were subjected to immunohistochemical analysis. Positive COX-2 staining yielded a brown-colored product: acetone (left), UVB alone (middle), and UVB plus H-89, 25 nmol (right). Percent of COX-2 positivity in epidermal layer was determined by counting the number of total COX-2 positive cells from 10 equal sections of immunostained tissues from each animal. *, *p*<0.001 (control versus UVB); **, *p*<0.01 (UVB versus 25 nmol H-89 plus UVB). (**C**) Cell lysates were analyzed for NOX-4 by using western blotting. *, *p*<0.001 (control versus UVB); **, *p*<0.01 (UVB versus 10 µmol DHA plus UVB); ***, *p*<0.01 (UVB versus 25 nmol H-89 plus UVB).

To determine the possible role of MSK1 in UVB-induced expression of COX-2 and NOX-4, we measured the expression of these proteins in UVB-irradiated mouse skin pretreated with H-89. As shown in Fig. 5A **,** topical application of H-89 (25 nmol/mouse) 40 min prior to UVB irradiation diminished the expression of COX-2 in mouse skin. Immunohistochemical analysis of COX-2 expression in mouse skin irradiated with UVB in the presence or absence of H-89 confirmed the inhibitory effect of H-89 on UVB-induced COX-2 expression (Fig. 5B). Pretreatment with H-89 also abolished NOX-4 expression **(** Fig. 5C **)** in UVB-irradiated mouse skin.

## Discussion

The health beneficial effects of ω-3 polyunsaturated fatty acids, in particular DHA, have been well demonstrated in a wide range of population-based and laboratory studies [32]. However, the molecular mechanisms underlying its anti-oxidative and anti-inflammatory activities have not been fully clarified. Solar radiation, particularly UVB, induces oxidative stress and inflammation in skin [2]. Aberrant expression of NOX [33] and COX-2 [34] plays a key role in inducing oxidative stress and inflammation. Thus, inhibition of NOX and COX-2 expression can protect against oxidative and inflammatory tissue damages, and hence can prevent carcinogenesis [24]. In the present study, we sought to examine the effect of UVB on the expression of COX-2 and NOX-4 in hairless mouse skin, and its possible modulation by DHA.

NOX comprises a group of enzymes, either membrane-bound or cytosolic, which mediates generation of ROS by diverse external stimuli [21]. Constitutive expression of NOX-1, NOX-2 and NOX-4 has been reported in human keratinocytes (HaCaT) [35]. Pharmacological inhibition of NOX inhibits phorbol ester-induced activation of p53 and induction of apoptosis in 7,12-dimethylbenz[*a*]anthracene-treated mouse skin [36]. However, little is known about the mechanism and consequences of NOX expression in mouse skin upon UVB exposure. Our study has revealed that the expression of NOX-4, one isoform of NOX enzymes, has been elevated in UVB-irradiated mouse skin as early as 1 h and persists until 6 h post-irradiation. NOX-4 has been reported to be induced by other stimuli, such as hypoxia [37], urotensin-II [38], transforming growth factor-β1 [39], angiotensin-II (Ang-II) [40] and TNFα [41]. The effect of UVB radiation on the expression of other isoforms of NOX in mouse skin merits further investigation. While the molecular mechanisms underlying UVB-induced NOX-4 expression have not been investigated earlier, UVB-induced COX-2 expression involves inappropriate amplification of cell signaling pathways comprising MAP kinases and redox-regulated transcription factors [15], [17].

DHA has been reported to inhibit NOX-4 expression in cultured cells stimulated with several stimuli other than UVB [42]. Our study provides the first demonstration that DHA can inhibit UVB-induced NOX-4 expression in mouse skin in vivo. The inhibitory effect of DHA on UVB-induced COX-2 expression in mouse skin is well correlated with several previous studies. It has been reported that DHA can inhibit expression of COX-2 in UV-stimulated keratinocytes [43]. An *et al.* also demonstrated that supplementation with DHA inhibited COX-2 expression in rat kidney undergoing partial nephroctomy [44]. In contrast, DHA enhanced phorbol ester- or IL-1β-induced COX-2 expression in rat vascular endothelial cells [45]. The opposing effects of DHA on COX-2 expression may be due to the stimulus- and cell type-specific response of the compound.

The presence of NF-κB-responsive *cis*-acting elements in the promoter regions of COX-2 [27] and NOX-4 [22] genes led us to examine the effects of DHA on UVB-induced NF-κB activation in hairless mouse skin. NF-κB, predominantly a heterodimer of p50 and p65 proteins, is normally sequestered in the cytoplasm by forming an inactive complex with the inhibitory protein IκBα [46]. Irradiation with UVB induced the phosphorylation of IKK and IκBα [19] and the induction of NF-κB [19] in hairless mouse skin. Our findings that topical application of DHA attenuated UVB-induced DNA binding of NF-κB, phosphorylation of IKKα/β, phosphorylation and degradation of IκBα, and nuclear localization of p50 and p65 proteins are in good agreement with the inhibitory effects of DHA on NF-κB activation in cultured cells exposed to stimuli other than UVB (53, 54). DHA has been shown to inhibit NF-κB activation in TNFα-treated human aortic endothelial cells [47] and lipopolysaccharide (LPS)-stimulated THP-1-derived macrophages [48]. Besides NF-κB, the transcriptional activation of NOX-4 [49] and COX-2 [50] is regulated by signal transducer and activator of transcription (STAT). Whether DHA can inhibit UVB-induced activation of STAT signaling as a mechanism underlying its inhibitory effects on the expression of COX-2 and NOX-4 in mouse skin needs additional investigation.

UVB irradiation induces phosphorylation of several upstream kinases, such as ERK [26], p38 MAP kinase [17] and Akt [15] in mouse skin. Activation of these upstream kinases leads to the amplification of NF-κB-mediated signaling and aberrant expression of COX-2 [20]. The inhibitory effects of DHA on UVB-induced phosphorylation of ERK and p38 MAP kinase, thus, provide a mechanistic basis of this ω-3 polyunsaturated fatty acid in suppressing COX-2 expression and NF-κB activation in mouse skin.

The inhibition of ERK has been shown to diminish NOX-4 expression in human aortic endothelial cells [42] and human monocyte-derived macrophages [51] stimulated with IL-1β and oxidized low density lipoprotein, respectively. In addition, transient overexpression of p65 induces the NOX-4 promoter activity in human aortic endothelial cells [22]. Thus, the inactivation of ERK and NF-κB signaling by DHA may partly account for its inhibition of UVB-induced NOX-4 expression. Whether the expression of NOX-4 in UVB-irradiated mouse skin is directly regulated by ERK and NF-κB is yet to be investigated.

Several studies have demonstrated that MSK1 functions as a signaling molecule downstream of ERK and p38 MAP kinase [23], [52]. Moreover, MSK1 plays a critical role in activating NF-κB through phosphorylation of p65 at the serine 276 residue [53]. Although MSK1 is phosphorylated at multiple sites, phosphorylation at the threonine 581 residue is essential for its catalytic activity [52]. We found that UVB irradiation induced phosphorylation of MSK1 at threonine 581 in mouse skin and that the treatment with DHA inhibited MSK1 phosphorylation. Treatment with H-89 failed to block phosphorylation of ERK and p38 MAP kinase, while U0126 and SB203580 attenuated phosphorylation of MSK1. These findings suggest that MSK1 acts as a downstream signaling molecule to ERK and p38 MAP kinase in UVB-irradiated mouse skin. We attempted to elucidate the role of MSK1 in the activation of NF-κB and subsequent expression of COX-2 and NOX-4 in mouse skin. The inhibition of the phosphorylation of IκBα nuclear localization of p50 and p65 phosphorylation of p65 and the DNA binding of NF-κB by pretreatment with H-89 suggest that MSK1 is a critical signaling molecule in UVB-induced activation of NF-κB in mouse skin. Moreover, the inhibitory effect of H-89 on UVB-induced NOX-4 and COX-2 expression indicates that MSK1 plays an important role in UVB-induced oxidative stress and inflammation in mouse skin.

As an ROS-generating enzyme NOX-4 may contribute to the activation of MAP kinases and NF-κB. Sedeek *et al.* demonstrated that the treatment of mouse proximal tubular cells with D-glucose induced the expression of NOX-4 and increased the phosphorylation of p38 MAP kinase. Inhibition of NOX-4 by GKT136901 abrogated D-glucose-induced p38 MAP kinase phosphorylation [54]. The induction of NOX-4 is also essential for LPS-induced activation of NF-κB in human embryonic kidney (HEK293T) cells [55]. Our observation that NOX-4 was induced in mouse skin at 1 h post-UVB irradiation raises the possibility that NOX-4 induction may contribute to UVB-induced activation of MAP kinases and NF-κB. However, the inhibition of UVB-induced phosphorylation of ERK and p38 MAP kinase and activation of NF-κB by DHA in mouse skin is unlikely to be mediated through its downregulation of NOX-4 expression, because pretreatment with DHA failed to inhibit UVB-induced NOX-4 expression at 1.5 h (data not shown), the time when DHA attenuated the activation of aforementioned MAP kinases and NF-κB.

Several studies have demonstrated that DHA undergoes enzymatic conversion by acetylated-COX-2 and 5-lipoxygenases (5-LOX) that generates a series of proresolving mediators responsible for early resolution of inflammation [56], [57]. We observed that DHA attenuated the activation of MAP kinases and NF-κB after UVB exposure for 1.5 h, the time when COX-2 was not induced in mouse skin (data not shown). 5-LOX is also an inducible enzyme that is expressed in human dermal fibroblasts after 24-h UVB exposure [58]. Thus, the inhibitory effect of DHA on UVB-induced expression of NOX-4 and COX-2 is unlikely to be mediated by its metabolites.

In conclusion, our study demonstrates that topical application of DHA protects against UVB-induced oxidative stress and inflammation through inactivation of NF-κB and downregulation of the upstream MSK1 signaling, which provides a mechanistic basis of anti inflammatory effects of DHA in mouse skin *in vivo*. Since oxidative stress and inflammation trigger tumor promotion, this study signifies the potential of DHA in preventing skin photocarcinogenesis.

## Materials and Methods

### Ethical statement

All experimental protocols were approved by the Animal Care and Use Committee (ACUC) of Seoul National University, South Korea (permit number: SNU-100504-3).

### Materials

DHA (purity>98%) was purchased from Cayman Chemical Co. (Ann Arbor, MI, USA). H-89, a pharmacological inhibitor of MSK1, was brought from Sigma-Aldrich (St Louis, MO, USA). U0126 and SB203580, selective inhibitors of ERK and p38 MAP kinase respectively, were procured from Tocris Bioscience (Ellisville, MO, USA). Primary antibodies for NOX-4, p65, p50, IκBα, pIKKα/β, ERK, pERK, p38, and MSK1 were supplied by Santa Cruz Biotechnology (Santa Cruz, CA, USA) and phospho-p65 (serine 276), pMSK1 (threonine 581) were procured from Cell Signaling Technology (Beverly, MA, USA). Antibodies for COX-2 and p38 MAP kinase were from Cayman Chemical Co. (Ann Arbor, MI, USA) and BD Bio-science Laboratories (San Jose, CA, USA), respectively. Anti-actin antibody was obtained from Sigma Chemical Company (St. Louis, MO, USA). Anti-rabbit and anti-mouse horseradish peroxidase-conjugated secondary antibodies were obtained from Zymed Laboratories Inc. (San Francisco, CA, USA). Enhanced chemiluminescent (ECL) detection kit and [γ-^32^P]ATP were purchased from Amersham Pharmacia Biotech (Buckinghamshire, UK). Oligonucleotide probe containing the NF-κB consensus sequence in mouse COX-2 promoter region was purchased from Promega (Madison, WI, USA). All other chemicals used were in the purest form available commercially.

### Animal treatment

Female HR-1 hairless mice (6–7 weeks age) were supplied from Sankyo Laboservice Corporation, Inc. (SLC, Tokyo, Japan). Animals were housed in climate-controlled quarters (24±1°C at 50% humidity) with a 12-h light/12-h dark cycle. DHA (2.5 and 10 µmol) was dissolved in 200 µl of acetone and applied topically to the dorsal skin 40 min before exposure to UVB (180 mJ/cm^2^) radiation.

### Source of UVB radiation

The source of UVB radiation was a 5×8 Watt tube, which emits an energy spectrum with high fluency in the UVB region (with a peak at 312 nm). A Biolink BLX-312 UV crosslinker (Vilbert Lourmat, Marne-la-Valée, France) was used in the present study to irradiate mouse skin.

### Western blot analysis

Dorsal skin of HR-1 hairless mice were treated with DHA (2.5 or 10 µmol/mouse), H-89 (25 or 100 nmol/mouse), SB203580 (5 µmol/mouse) or U0126 (5 µmol/mouse) 40 min before exposure to UVB (180 mJ/cm^2^) and sacrificed by cervical dislocation either 1.5 or 6 h later. Control animals were treated with vehicle only. For the preparation of mouse epidermal protein extract, fat and dermis were removed from the harvested skin samples by keeping on ice, and the fat-free epidermis was immediately placed in liquid nitrogen and pulverized in mortar. The pulverized skin was homogenized on ice for 20 s with a polytron tissue homogenizer and lysed in 1 ml ice-cold lysis buffer (150 mM NaCl, 0.5% Triton X-100, 50 mM Tris-HCl pH 7.4, 20 mM EGTA, 1 mM dithiothreitol, 1 mM Na_3_VO_4_ and protease inhibitor cocktail tablet). Lysates were centrifuged at 14,800 x *g* for 15 min. The supernatant was collected and total protein concentration was quantified by using the bicinchoninic acid (BCA) protein assay kit. Cell lysates (30 to 50 µg protein) were boiled in sodium dodecyl sulfate (SDS) sample loading buffer for 5 min before electrophoresis on 10–12% SDS-polyacrylamide gel. After transfer to polyvinylidene difluoride membrane, the blots were blocked with 5% fat-free dry milk-PBST (phosphate-buffer saline containing 0.1% Tween 20) or 1% BSA in TBST (Tris-buffer saline containing 0.1% Tween 20) for 1 h at room temperature and then washed with PBST or TBST buffer. The membranes were incubated for 2 h at room temperature with 1:1000 dilutions of primary antibodies for actin, ERK and p38, for 12 h at 4°C with 1:1000 dilutions of primary antibodies for phospho-p38, phospho-ERK, IκBα, phospho-IκBα, COX-2, NOX-4, pIKKα/β, MSK1, pMSK1, p50 and p65. Blots were washed three times with PBST or TBST at 5 min intervals followed by incubation with 1:5000 dilution of respective horseradish peroxidase (HRP) conjugated secondary antibodies (rabbit, goat or mouse) in 3% fat-free dry milk-PBST for 1 h at room temperature. The blots were rinsed again three times with PBST or TBST. The immunoblots were visualized with an ECL detection kit according to the manufacturer's instructions.

### Immunohistochemical analysis

The dissected mouse skin from different treatment groups was prepared for immunohistochemical analysis of the expression of COX-2 and phospho-MSK1 (threonine 581). Four-micrometer sections of 10% formalin-fixed, paraffin-embedded tissues were cut on salinized glass slides and deparaffinized three times with xylene, and rehydrated through graded alcohol bath. The deparaffinized sections were heated with microwave and boiled twice for 6 min in 10 mM citrate buffer (pH 6.0) for antigen retrieval. To diminish non-specific staining, each section was treated with 3% hydrogen peroxide and 4% peptone casein blocking solution for 15 min. For the detection of respective protein expression, slides were incubated with affinity purified rabbit polyclonal anti-COX-2 and anti-pMSK1 (threonine 581) (1:50) at room temperature for 40 min in Tris-buffered saline containing 0.05% Tween 20 and then developed using anti-rabbit HRP EnVision^TM^ System (Dako, Glostrup, Denmark). The peroxidase binding sites were detected by staining with 3,3′-diaminobenzidine tetrahydrochloride (Dako, Glostrup, Denmark). Finally, counterstaining was performed using Mayer's hematoxylin.

### Preparation of cytosolic and nuclear extracts

The cytosolic and nuclear extract from mouse skin was prepared as described previously [19]. In brief, scraped dorsal skin of mice was homogenized in 800 µl of hypotonic buffer A [10 mM HEPES (pH 7.8), 10 mM KCl, 2 mM MgCl_2_, 1 mM dithiothreitol, 0.1 mM EDTA, 0.1 mM phenylmethylsulfonylfluoride (PMSF)]. To the homogenates was added 80 µl of 10% Nonidet P-40 (NP-40) solution, and the mixture was then centrifuged for 2 min at 14,000 x *g*. The supernatant was collected as cytosolic fraction. The precipitated nuclei were washed once with 500 µl of buffer A plus 40 µl of 10% NP-40, centrifuged, resuspended in 200 µl of buffer C [50 mM HEPES (pH 7.8), 50 mM KCl, 300 mM NaCl, 0.1 mM EDTA, 1 mM dithiothreitol, 0.1 mM PMSF, 20% glycerol] and centrifuged for 5 min at 14,800 x *g*. The supernatant containing nuclear proteins was collected and stored at −70°C after determination of protein concentration.

### Electrophoretic mobility shift assay (EMSA)

The EMSA for DNA binding of NF-κB was performed using a DNA-protein binding detection kit (Gibco BRL, Grand Island, NY, USA), according to the manufacturer's protocol. Briefly, oligonucleotide harboring the binding site for NF-κB (5′–GATCGAGGGGGACTTTCCCAGC- 3′) was labeled with [γ-^32^P]ATP by T4 polynucleotide kinase (Takara Bio Inc., Shiga, Japan) and purified on a Nick column (Amersham Pharmacia Biotech, USA). The binding reaction was carried out in 25 µl of mixture containing 5 µl of incubation buffer [10 mM Tris-HCl (pH 7.5), 100 mM NaCl, 1 mM dithiothreitol, 1 mM EDTA, 4% glycerol, and 0.1 mg/ml sonicated salmon sperm DNA], 10 µg of nuclear extracts, and 100000 cpm of [γ-^32^P]ATP-end labeled oligonucleotide. After 50 min incubation at room temperature, 2 µl of 0.1% bromophenol blue was added, and samples were electrophoresed through 6% nondenaturing polyacrylamide gel at 150 V in a cold room for 2 h. To ensure the specificity of the binding, a competition assay was carried out with the excess unlabeled oligonucleotide. Finally, the gel was dried and exposed to an X-ray film.

### Statistical analysis

Values were expressed as the mean ± SEM of at least three independent experiments. Statistical significance was determined by Student's *t* test and a *p*-value of less than 0.05 was considered to be statistically significant.

## Supporting Information

Figure S1 **Statistical analysis of data presented in** **Fig. 3A, 3B, 3C, 3E, 3F and 3G** **.** Band intensity of immunoblots obtained from three mice in a treatment group was subjected to densitometric analysis by using GelPro 3.0 and a ratio of band intensity of respective immunoblots to that of internal standard actin or Lamin B was calculated. Statistical analysis was performed using SigmaPlot 2001 to determine the level of significance. (Figure S1A) time-dependent expression of pMSK1 in UVB-irradiated mouse skin. *, *p*<0.001 (control versus UVB). (Figure S1B) Inhibitory effect of H-89 on UVB-induced phosphorylation of MSK1 in mouse skin. *, *p*<0.001 (control versus UVB); **, *p*<0.001 (UVB versus 25 nmol H-89 plus UVB); ***, *p*<0.01 (UVB versus 100 nmol H-89 plus UVB). (Figure S1C) pretreatment with DHA attenuated UVB-induced phosphorylation of MSK1 in mouse skin. *, *p*<0.001 (control versus UVB); **, *p*<0.05 (UVB versus 10 µmol DHA plus UVB). (Figure S1D) Kinetics of UVB-induced phosphorylation of ERK and p38 MAP kinase in mouse skin. *, *p*<0.001 (control versus UVB). (Figure S1E) Inhibitory effects of DHA on UVB-induced phosphorylation of ERK and p38 MAP kinase. *, *p*<0.001 (control versus UVB); **, *p*<0.05 (UVB versus 2.5 µmol DHA plus UVB); ***, *p*<0.001 (UVB versus 10 µmol DHA plus UVB). (Figure S1F) Effect of H-89 on UVB-induced phosphorylation of ERK and p38 MAP kinase. *, *p*<0.001 (control versus UVB alone).
(TIF)

Figure S2 **Effect of H-89 on UVB-induced phosphorylation of IKKα/β in mouse skin.** Topical application of H-89 (25 nmol) failed to alter UVB-induced phosphorylation of IKKα/β in mouse skin.
(TIF)
