# Supplementary figures and images for: Docosahexaenoic Acid Inhibits UVB-Induced Activation of NF-κB and Expression of COX-2 and NOX-4 in HR-1 Hairless Mouse Skin by Blocking MSK1 Signaling
Source: PLoS One. 2011 Nov 28;6(11):e28065. doi: 10.1371/journal.pone.0028065 (PMC3225387; doi:10.1371/journal.pone.0028065)

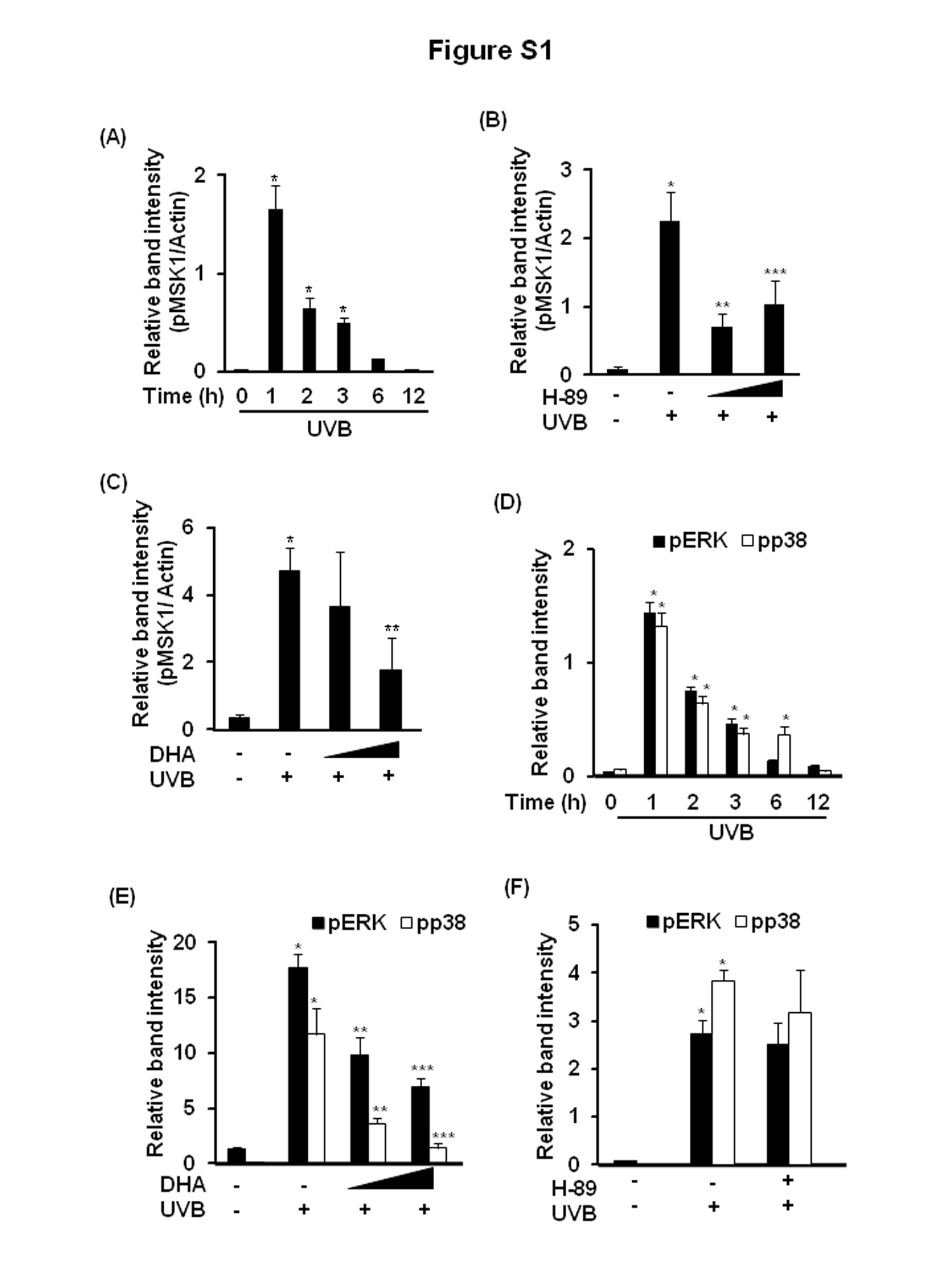

Supplement: Figure S1 — Statistical analysis of data presented in Fig. 3A, 3B, 3C, 3E, 3F and 3G . Band intensity of immunoblots obtained from three mice in a treatment group was subjected to densitometric analysis by using GelPro 3.0 and a ratio of band intensity of respective immunoblots to that of internal standard actin or Lamin B was calculated. Statistical analysis was performed using SigmaPlot 2001 to determine the level of significance. (Figure S1A) time-dependent expression of pMSK1 in UVB-irradiated mouse skin. *, p<0.001 (control versus UVB). (Figure S1B) Inhibitory effect of H-89 on UVB-induced phosphorylation of MSK1 in mouse skin. *, p<0.001 (control versus UVB); **, p<0.001 (UVB versus 25 nmol H-89 plus UVB); ***, p<0.01 (UVB versus 100 nmol H-89 plus UVB). (Figure S1C) pretreatment with DHA attenuated UVB-induced phosphorylation of MSK1 in mouse skin. *, p<0.001 (control versus UVB); **, p<0.05 (UVB versus 10 µmol DHA plus UVB). (Figure S1D) Kinetics of UVB-induced phosphorylation of ERK and p38 MAP kinase in mouse skin. *, p<0.001 (control versus UVB). (Figure S1E) Inhibitory effects of DHA on UVB-induced phosphorylation of ERK and p38 MAP kinase. *, p<0.001 (control versus UVB); **, p<0.05 (UVB versus 2.5 µmol DHA plus UVB); ***, p<0.001 (UVB versus 10 µmol DHA plus UVB). (Figure S1F) Effect of H-89 on UVB-induced phosphorylation of ERK and p38 MAP kinase. *, p<0.001 (control versus UVB alone). (TIF) [file pone.0028065.s001.tif]

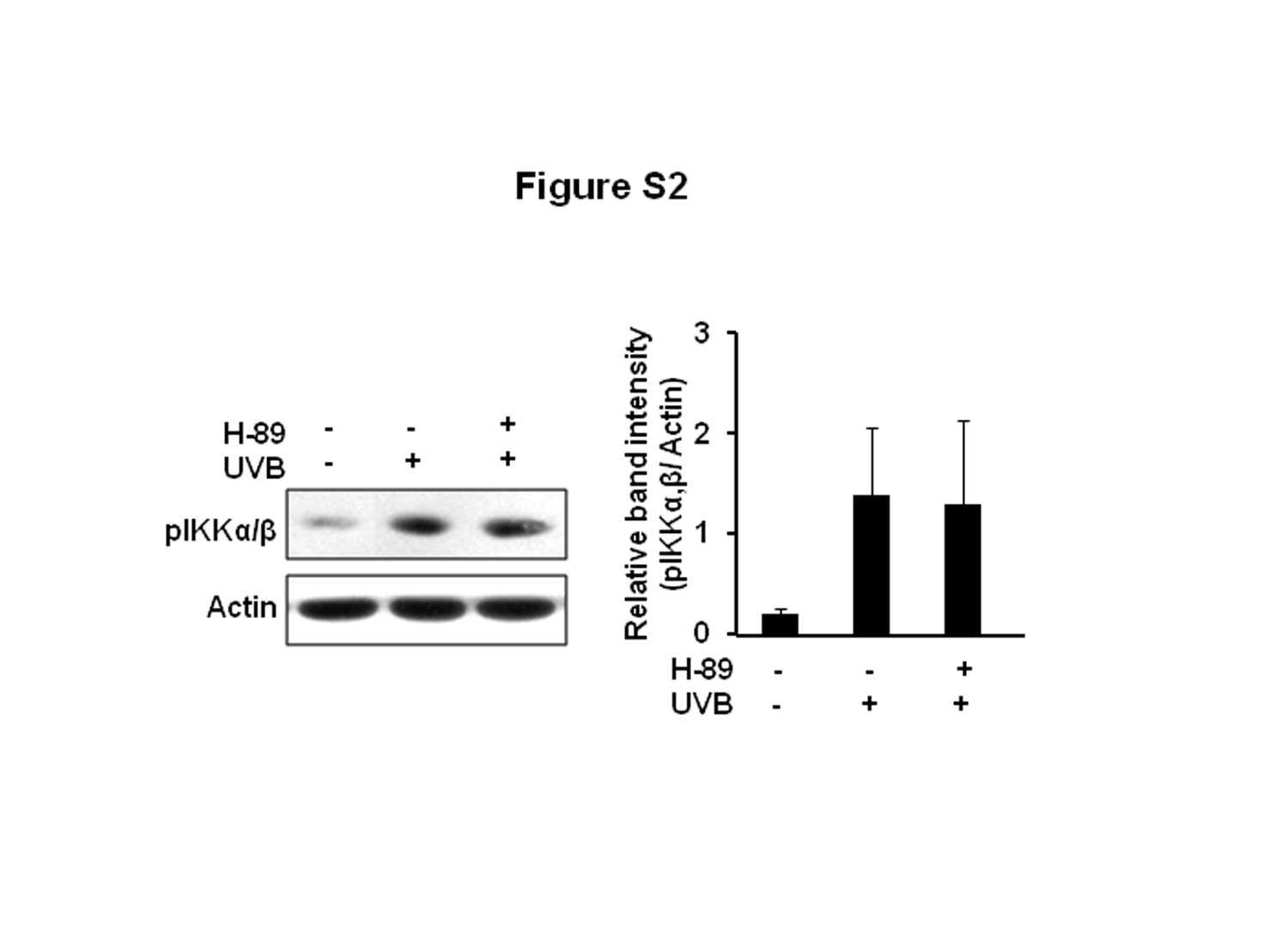

Supplement: Figure S2 — Effect of H-89 on UVB-induced phosphorylation of IKKα/β in mouse skin. Topical application of H-89 (25 nmol) failed to alter UVB-induced phosphorylation of IKKα/β in mouse skin. (TIF) [file pone.0028065.s002.tif]
